# Membrane-Mediated Cooperative Interactions of CD47 and SIRP*α*

**DOI:** 10.3390/membranes13110871

**Published:** 2023-11-02

**Authors:** Long Li, Chen Gui, Jinglei Hu, Bartosz Różycki

**Affiliations:** 1 Kuang Yaming Honors School, Nanjing University, Nanjing 210023, China; lilong@lnm.imech.ac.cn (L.L.); mg21980003@smail.nju.edu.cn (C.G.); hujinglei@nju.edu.cn (J.H.); 2 State Key Laboratory of Nonlinear Mechanics and Beijing Key Laboratory of Engineered Construction and Mechanobiology, Institute of Mechanics, Chinese Academy of Sciences, Beijing 100190, China; 3 Institute of Physics, Polish Academy of Sciences, Aleja Lotników 32/46, 02-668 Warsaw, Poland

**Keywords:** membrane adhesion, biomimetics, computer simulations, CD47, SIRP*α*

## Abstract

The specific binding of the ubiquitous ‘marker of self’ protein CD47 to the SIRPα protein anchored in the macrophage plasma membrane results in the inhibition of the engulfment of ‘self’ cells by macrophages and thus constitutes a key checkpoint of our innate immune system. Consequently, the CD47–SIRPα protein complex has been recognized as a potential therapeutic target in cancer and inflammation. Here, we introduce a lattice-based mesoscale model for the biomimetic system studied recently in fluorescence microscopy experiments where GFP-tagged CD47 proteins on giant plasma membrane vesicles bind to SIRPα proteins immobilized on a surface. Computer simulations of the lattice-based mesoscale model allow us to study the biomimetic system on multiple length scales, ranging from single nanometers to several micrometers and simultaneously keep track of single CD47–SIRPα binding and unbinding events. Our simulations not only reproduce data from the fluorescence microscopy experiments but also are consistent with results of several other experiments, which validates our numerical approach. In addition, our simulations yield quantitative predictions on the magnitude and range of effective, membrane-mediated attraction between CD47–SIRPα complexes. Such detailed information on CD47–SIRPα interactions cannot be obtained currently from experiments alone. Our simulation results thus extend the present understanding of cooperative effects in CD47–SIRPα interactions and may have an influence on the advancement of new cancer treatments.

## 1. Introduction

The adhesion of cell membranes arises from the specific binding of membrane-anchored receptor proteins to their ligands anchored in the apposing membrane. This receptor–ligand binding at the cell interface is essential for various biological processes, including tissue formation, immune responses, and signaling. An immunologically relevant example is the binding of the ubiquitous ‘marker of self’ protein CD47 to the SIRPα protein anchored in the plasma membranes of macrophages. The CD47–SIRPα binding results in the inhibition of the engulfment of ‘self’ cells by macrophages and thus constitutes a checkpoint of our innate immune system [1]. The binding of CD47 to SIRPα has been found to play important roles in phagocytosis, auto-immunity, and host defense [1,2]. As such, the CD47–SIRPα protein complex has been recognized as a potential therapeutic target in cancer [3,4] and inflammation [5].

The strength of the binding of molecules in a solution is typically quantified by the equilibrium constant $K_{3D}={\left[\mathrm{RL}\right]}_{3D}/{\left[R\right]}_{3D}{\left[L\right]}_{3D}$, where ${\left[\mathrm{RL}\right]}_{3D}$ is the volume concentration of molecular complexes, whereas ${\left[R\right]}_{3D}$ and ${\left[L\right]}_{3D}$ are the volume concentrations of free molecules in the solution. It is often assumed by analogy that the strength of the binding of membrane-anchored molecules is captured via the two-dimensional binding constant $K_{2D}=\left[\mathrm{RL}\right]/\left[R\right]\left[L\right]$, where [RL] is the area concentration of receptor–ligand complexes, whereas [R] and [L] are the area concentrations of the free receptors and free ligands, respectively. A major difference between $K_{3D}$ and $K_{2D}$ is that the receptor–ligand binding that mediates membrane adhesion is determined not only with direct interactions between the receptor molecule and its ligand but also with the elastic properties of the adhering membranes [6,7]. In particular, for relatively weak adhesion and flexible membranes, $K_{2D}$ has been shown to be inversely proportional to the relative roughness $\xi_{⊥}$ of the membrane surfaces brought about by thermal fluctuations [6].

**Figure 1:**
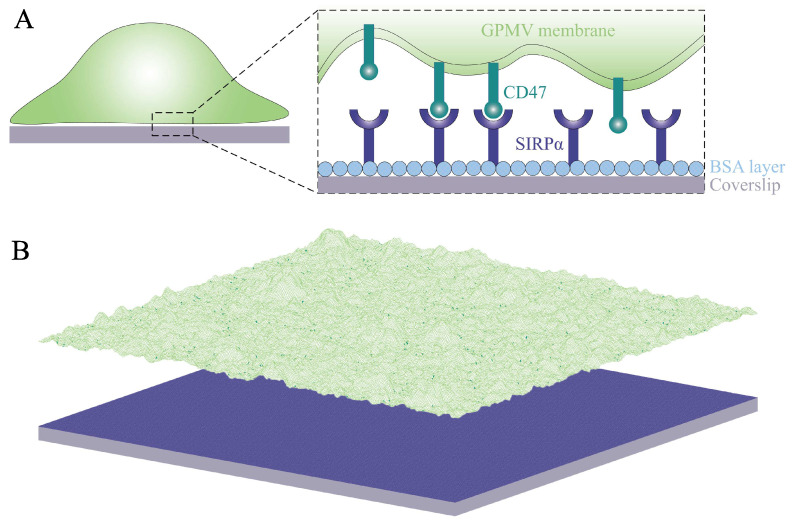
(**A**) Cartoon of the system under study: SIRPα receptors (dark blue) immobilized on a planar surface (gray) can bind CD47 ligands (dark green) on a GPMV (light green). The surface is coated with BSA (light blue) to prevent non-specific adhesion of the membrane to the surface. (**B**) Lattice-based mesoscale model that takes into account (i) diffusion of the membrane-anchored ligands, (ii) binding and unbinding of the receptors and their ligands, and (iii) elastic deformations and thermal undulations of the membrane. The color code is as in panel A. The lateral size of the system is 6 μm.

The relationship $K_{2D}∼1/\xi_{⊥}$ derived by Hu et al. [6] is associated with a positive cooperativity in the receptor–ligand binding process, which can be explained as follows: Fluid membranes are rather soft and undergo thermally excited fluctuations. The receptor–ligand complex formation suppresses membrane fluctuations and causes the average distance between the membranes to be closer to the optimal distance for the receptor–ligand binding, which in turn facilitates the formation of additional receptor–ligand complexes between the two membranes. The feedback between the suppression of membrane fluctuations and the formation of receptor–ligand complexes leads to an effect of membrane-mediated binding cooperativity. This cooperativity effect has been predicted theoretically [8], examined in dissipative particle dynamics (DPD) simulations of a generic coarse-grained model [6], and confirmed quantitatively in fluorescence microscopy experiments with GFP-tagged CD47 on giant plasma membrane vesicles (GPMVs) binding to SIRPα immobilized on a surface [9] (Figure 1A).

The adhesion of cell membranes involves multiple length scales ranging from Angstroms to micrometers. Namely, the specific binding of the receptor proteins to their ligands occurs on the Angstrom length scale. The thickness of the lipid membrane is about 5 nanometers. The extension of the extracellular domains of the receptor and ligand proteins is typically of the order of 10 nanometers. The typical distance between the receptor–ligand complexes involved, e.g., in immune responses or signaling is of the order of 100 nanometers. Finally, the linear extension of the interface between cell membranes is of the order of micrometers. Because all of these length scales are relevant, theoretical and computational studies on membrane adhesion are challenging and require multi-scale modeling and suitable approximations that capture the essential physics of the system under study. Here, we use a lattice-based mesoscale model that captures the relevant length scales [10,11,12,13]. In particular, it takes into account (i) the diffusion of the membrane-anchored adhesion molecules, (ii) the binding and unbinding of the receptors and their ligands, and (iii) the elastic deformations and thermal undulations of fluid lipid membranes. We adapt this model to carry out large-scale simulations of the SIRPα–CD47 system studied in the fluorescence microscopy experiments [9]. The system under study and the model in use are illustrated in Figure 1. Our simulations not only reproduce several different experiments [1,9,14] but also yield quantitative predictions on the range and magnitude of fluctuation-induced, membrane-mediated attraction between CD47–SIRPα complexes. Since the SIRPα–CD47 protein complex has been recognized as a promising therapeutic target in cancer [3,4], our detailed studies on the cooperative binding of CD47 to SIRPα may have an influence on the advancement of new cancer treatments.

## 2. Model and Methods

We model the system used in the fluorescence microscopy experiments, where GST-tagged SIRPα molecules (receptors) immobilized on a planar surface bind GFP-labeled CD47 molecules (ligands) on a GPMV [9], as illustrated in Figure 1A. The surface is coated with BSA to prevent non-specific adhesion of the GPMV membrane to the surface. The area concentration of the receptors on the substrate is [R]≈4000/μ$m^{2}$, which provides a characteristic length scale $a=1/\sqrt{\left[R\right]}\approx 15$ nm.

The model is based on representing the GPMV membrane as a two-dimensional elastic sheet and discretizing this sheet into ‘patches’ of linear size *a* larger than the membrane thickness [8,15,16], as illustrated in Figure 1B. The choice of the patch size *a* is somewhat ambiguous. One option is to take a=5 nm to capture the complete spectrum of the bending deformations of fluid membranes [8,17]. Another option is to take a somewhat larger patch size, a=10 nm, to match it to an average exclusion radius of membrane proteins [12,18]. For comparison, Weikl and Lipowsky used a=70 nm in their studies on pattern formation during T-cell adhesion [19]. Here, we take a=15 nm to have, on average, one surface-immobilized receptor per membrane patch.

Membrane patches are labeled with index $i=(i_{x},i_{y})$, which is a set of two integer numbers that specify the Cartesian coordinates in a reference plane. Here, we take the reference plane to coincide with the planar surface coated with BSA. The distance between membrane patch *i* and the BSA-coated surface is denoted by $l_{i}$. The configuration of the membrane is thus given by a set $l=\{l_{i}\}$.

The spatial distribution of ligands on the membrane is described using a set $n=\{n_{i}\}$ of binary variables with $n_{i}=0$ or $n_{i}=1$ indicating, respectively, the absence or presence of a ligand at membrane patch *i*. It is assumed at this point that any patch can accommodate only one ligand, which is in contrast with the model for pattern formation during T-cell adhesion [19], where multiple adhesion proteins could occupy a single patch. It should be noted, however, that the patch size *a* used in that model is about 5 times larger than in our present model.

To ensure the specific receptor–ligand binding, one ligand on membrane patch *i* only binds one receptor if $l_{i}$ is within a certain binding range, namely, $l_{b}-\frac{1}{2}l_{\mathrm{we}}<l_{i}<l_{b}+\frac{1}{2}l_{\mathrm{we}}$. We define parameter $l_{b}=l_{\mathrm{CD}47–\mathrm{SIRP}\alpha }-l_{\mathrm{BSA}}$, where $l_{\mathrm{CD}47–\mathrm{SIRP}\alpha }$ denotes the length of the receptor–ligand complex and $l_{\mathrm{BSA}}$ denotes the thickness of the BSA layer on the surface. Parameter $l_{\mathrm{we}}$ reflects the flexibility of the receptor–ligand complex. Here, $l_{\mathrm{we}}\approx 1.2$ nm, as determined in molecular simulations of the surface-immobilized GST-tagged SIRPα in complex with the membrane-anchored GFP-labeled CD47 [9].

The total energy of receptor–ligand interactions reads

(1) $$H_{\mathrm{int}}\left\{l,n\right\}=\sum_{i}n_{i}V\left(l_{i}\right)$$

where the sum is performed over all membrane patches and the receptor–ligand binding potential

(2) $$V\left(l_{i}\right)=-U\Theta \left(\frac{l_{\mathrm{we}}}{2}-\left|l_{i}-l_{b}\right|\right)$$

is a square-well potential of depth *U*, width $l_{\mathrm{we}}$, and range $l_{b}$. Here, Θ denotes the Heaviside step function, i.e., Θ(x)=1 if x>0 and Θ(x)=0 otherwise. The depth *U* of potential $V(l_{i})$ can be interpreted as the receptor–ligand binding energy.

The three-dimensional binding constant $K_{3D}$ of the soluble variants of CD47 and SIRPα has been determined experimentally, yielding the dissociation constant $1/K_{3D}\approx 1\;\mu M$ [1]. An estimate for the binding energy *U* can be obtained from the relation $K_{3D}=a^{2}\;l_{\mathrm{we}}\;e^{U/k_{B}T}$, where $k_{B}$ and *T* denote the Boltzmann constant and room temperature, respectively. Taking a=15 nm, $l_{\mathrm{we}}=1.2$ nm, and $1/K_{3D}=1\;\mu M$, we obtain $U\approx 9k_{B}T$.

In addition to the receptor–ligand interaction energy, $H_{\mathrm{int}}$, the Hamiltonian of the system under study also comprises the energy of membrane bending. We adapt the Helfrich theory for membrane elasticity [20] and use the formula derived by Weikl and Lipowsky [21,22] to compute the energy of membrane bending

(3) $$H_{\mathrm{el}}\left\{l\right\}=\frac{\kappa }{2a^{2}}\sum_{i}{\left(\Delta_{d}l_{i}\right)}^{2}\;.$$

Here, κ is the bending rigidity modulus of the membrane and $\Delta_{d}l_{i}$ denotes a discretized Laplacian which is equal to twice the local mean curvature of the membrane surface times $a^{2}$ [23]. It is implicitly assumed here that the membrane is not under tension and has no spontaneous curvature. The bending rigidity modulus of the GPMV membrane has been determined experimentally using flicker spectroscopy, leading to $\kappa \approx 10\;k_{B}T$ [9].

It should be noted that unspecific membrane–surface interactions (i.e., interactions not related to the specific receptor–ligand binding) are not included in the model, except for the short-ranged steric repulsion between the membrane and the BSA-coated surface, which is taken into account using a constraint $l_{i}>0$. This assumption can be justified because the GPMVs in the fluorescence microscopy experiments have been observed not to adhere to the BSA-coated surface in the absence of the GST-tagged SIRPα molecules immobilized on the surface [9].

### 2.1. Monte Carlo Simulations

Monte Carlo (MC) simulations were performed with Hamiltonian $H\left\{l,n\right\}=H_{\mathrm{el}}\left\{l\right\}+H_{\mathrm{int}}\left\{l,n\right\}$ in the canonical ensemble, where the temperature *T*, the number of membrane patches, the number $N_{R}$ of immobile receptors, and the number $N_{L}$ of mobile ligands were fixed. Periodic boundary conditions were applied in the directions parallel to the planar surface. Two types of trial moves were used: (i) vertical local displacements of single patches to take into account deformations and thermal undulations of the membrane and (ii) horizontal shifts of single ligands to capture their diffusion within the membrane. The trial moves of type (i) caused variations in the field *l* of local distances between the membrane and the BSA-coated surface. Here, the maximal displacement of any membrane patch was 1.5 nm. In the trial moves of type (ii), the ligands were attempted to hop between neighboring patches, which led to variations in the composition field *n*. All of the trial moves were accepted according to the standard Metropolis criterion. Any trial move of type (i) leading to $l_{i}\leq 0$ was rejected to prevent any overlaps of the membrane with the BSA-coated surface.

The proportion of trial moves (i) and (ii) was chosen according to physical time scales as in our earlier works [12,24]. In one MC cycle, on average, all of the membrane patches were attempted to be vertically displaced ten times, whereas all of the ligands were attempted to be horizontally shifted once. Each of the MC simulation runs comprised $6\times 10^{7}$ MC cycles, where the initial $10^{7}$ cycles were used for equilibration and the subsequent $5\times 10^{7}$ cycles for data acquisition.

In the fluorescence experiments [9], the area concentration [RL] of receptor–ligand complexes was in the range between about 35 and 85 CD47–SIRPα complexes per μ$m^{2}$. We performed the MC simulations in the same range of concentrations. The membrane in the MC simulations was composed of 400×400 square patches, corresponding to an area of the adhesion zone of 36 μ$m^{2}$. We thus performed a series of simulations with the total number of ligands $N_{L}=1080,\;1440,\;1800,\;2160,\;2520,\;2880$, and 3240, corresponding to an area concentration of ligands between 30 and 90 per μ$m^{2}$.

Molecular modeling shows that the complex of surface-immobilized GST-tagged SIRPα and the membrane-anchored GFP-labeled CD47 has an average length $l_{\mathrm{CD}47–\mathrm{SIRP}\alpha }\approx 17$ nm [9]. The thickness $l_{\mathrm{BSA}}$ of the BSA layer has not been determined. Thus, the exact value of parameter $l_{b}=l_{\mathrm{CD}47–\mathrm{SIRP}\alpha }-l_{\mathrm{BSA}}$ is unknown. Therefore, we performed a series of simulations with $l_{b}=5.4,\;6.6,\;7.8,\;9,$ and 10.2 nm.

In summary, we performed the MC simulations with the following values of the model parameters: a=15 nm, $\left[R\right]=1/a^{2}$, $\kappa =10\;k_{B}T$, $U=9\;k_{B}T$, $l_{\mathrm{we}}=1.2$ nm, $l_{b}=5.4,6.6,7.8,9,10.2$ nm, and $N_{L}=1080,\;1440,\;1800,\;2160,\;2520,\;2880,\;3240$. We determined the average area concentration [L] of free ligands, the average area concentration [RL] of receptor–ligand complexes, and, hence, the two-dimensional binding affinity

(4) $$K_{2D}=\frac{\left[\mathrm{RL}\right]}{\left[R][L\right]}\;.$$

We also measured the average distance $\langle l_{i}\rangle$ between the membrane and the BSA-coated surface as well as the membrane roughness

(5) $$\xi_{⊥}={\left(\langle l_{i}^{2}\rangle -{\langle l_{i}\rangle }^{2}\right)}^{1/2}$$

where the angular brackets denote the ensemble average. The roughness is caused by the thermal fluctuations of the membrane.

### 2.2. Analysis of Binding Kinetics

We adapt the maximum likelihood method developed by Hu et al. [6] for extracting the binding kinetics from DPD trajectories [6]. We apply this method to the MC trajectories of the CD47–SIRPα system under study. The receptor–ligand binding and unbinding events divide any trajectory into different states with different numbers of receptor–ligand complexes. A system with $N_{R}$ receptors and $N_{L}$ ligands has (N+1) states in total, where $N=\min(N_{R},\;N_{L})$ is the maximum number of receptor–ligand complexes. Thus, a given trajectory can be mapped to a Markov model

(6) $$\begin{array}{c} 0\overset{k_{+}^{\left(0\right)}}{\underset{k_{-}^{\left(1\right)}}{⇌}}1\overset{k_{+}^{\left(1\right)}}{\underset{k_{-}^{\left(2\right)}}{⇌}}2\overset{k_{+}^{\left(2\right)}}{\underset{k_{-}^{\left(3\right)}}{⇌}}3\cdots N-1\overset{k_{+}^{\left(N-1\right)}}{\underset{k_{-}^{\left(N\right)}}{⇌}}N \end{array}$$

where the transition rates $k_{+}^{\left(n\right)}$ and $k_{-}^{\left(n\right)}$ are related, respectively, to the on- and off-rate constants $k_{\mathrm{on}}^{\left(n\right)}$ and $k_{\mathrm{off}}^{\left(n\right)}$ via

(7) $$k_{+}^{\left(n\right)}=\frac{1}{A}\left(N_{L}-n\right)\left(N_{R}-n\right)k_{\mathrm{on}}^{\left(n\right)}$$

and

(8) $$k_{-}^{\left(n\right)}=nk_{\mathrm{off}}^{\left(n\right)}$$

where *A* denotes the area of the contact zone.

The on- and off-rate constants $k_{\mathrm{on}}^{\left(n\right)}$ and $k_{\mathrm{off}}^{\left(n\right)}$ in Equations (7) and (8) can be determined from the observed number of transitions between the states and from the overall dwell times in the states. The binding and unbinding events divide a given trajectory into time windows *j* of length $t_{j}$ in state $n_{j}$, which are followed by a transition into state $n_{j}+s_{j}$ with $s_{j}=1$ or $s_{j}=-1$. The probability for staying in state $n_{j}$ for a dwell time $t_{j}$ is $P_{n_{j}}\left(t_{j}\right)=\exp\left\{-\left[k_{+}^{\left(n_{j}\right)}+k_{-}^{\left(n_{j}\right)}\right]t_{j}\right\}$. The probability for the time window *j* with the observed transition is $p_{j}\propto P_{n_{j}}\left(t_{j}\right)\cdot k_{+}^{\left(n_{j}\right)}$ for $s_{j}=1$ and $p_{j}\propto P_{n_{j}}\left(t_{j}\right)\cdot k_{-}^{\left(n_{j}\right)}$ for $s_{j}=-1$. The likelihood function is the probability of the whole trajectory and takes the form

(9) $$\begin{array}{c} L=\prod_{j}p_{j}=\prod_{n=0}^{N}{\left[k_{+}^{\left(n\right)}\right]}^{N_{n}^{+}}{\left[k_{-}^{\left(n\right)}\right]}^{N_{n}^{-}}e^{-\left[k_{+}^{\left(n\right)}+k_{-}^{\left(n\right)}\right]T_{n}} \end{array}$$

where $N_{n}^{+}$ is the total number of transitions from state *n* to n+1, $N_{n}^{-}$ the total number of transitions from state *n* to n−1, and $T_{n}$ the total dwell time in state *n*.

Maximizing the likelihood function *L* in Equation (9) with respect to the rate constants $\left\{k_{\mathrm{on}}^{\left(n\right)}\right\}$ and $\left\{k_{\mathrm{off}}^{\left(n\right)}\right\}$ leads to the maximum likelihood estimators

(10) $$k_{\mathrm{on}}^{\left(n\right)}=\frac{N_{n}^{+}A}{\left(N_{R}-n\right)\left(N_{L}-n\right)T_{n}}$$

and

(11) $$k_{\mathrm{off}}^{\left(n\right)}=\frac{N_{n}^{-}}{nT_{n}}\;.$$

In each simulation, we record the numbers of transitions $N_{n}^{+}$ and $N_{n}^{-}$ as well as the overall dwell times in each state $T_{n}$, and then we estimate the binding rate constants in each state according to Equations (10) and (11).

For *n* around the average number of receptor–ligand complexes, $\bar{n}$, the values of $k_{\mathrm{on}}^{\left(n\right)}$ and $k_{\mathrm{off}}^{\left(n\right)}$ hardly change with *n*. We thus define the association rate constants $k_{\mathrm{on}}=k_{\mathrm{on}}^{\left(\bar{n}\right)}$ and the dissociation rate constants $k_{\mathrm{off}}=k_{\mathrm{off}}^{\left(\bar{n}\right)}$. The binding affinity given by Equation (4) is then consistent with $K_{2D}=k_{\mathrm{on}}/k_{\mathrm{off}}$.

The dwell times $T_{n}$ in Equations (10) and (11) are in units of the number of MC steps. To relate one MC step to the physical time, τ, we follow Weikl and Lipowsky [19] and use the two-dimensional diffusion relation $D=a^{2}/\tau$, where *D* is the diffusion coefficient of membrane proteins. Taking D≈1μm${}^{2}$/s and a=15 nm, we obtain τ≈60μs, which implies that each of the simulations comprising $6\times 10^{7}$ MC cycles corresponds to the physical time of about one hour.

## 3. Results

**Figure 2:**
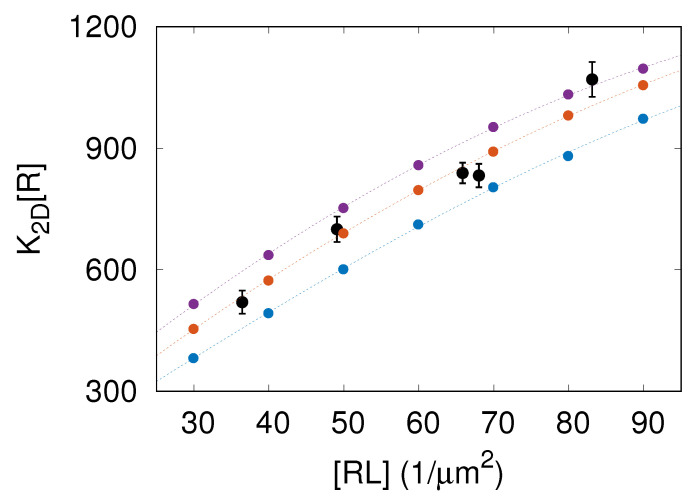
Two-dimensional binding affinity $K_{2D}$ times the area concentration of receptors, [RL]=4000/μm${}^{2}$, versus the average area concentration [RL] of receptor–ligand complexes. The data points in black correspond to the experimental FRAP data taken from Figure 2A in Reference [9]. The points in blue, orange, and purple represent the results of MC simulations with $l_{b}=5.4,\;6.6$, and 7.8 nm, respectively. The dashed lines are to guide the eye.

We performed MC simulations of the model introduced in Section 2. We measured the area concentration [RL] of receptor–ligand complexes and the two-dimensional binding affinity $K_{2D}$ as given by Equation (4). The results of these simulations with $l_{b}=5.4,\;6.6$, and 7.8 nm are shown in Figure 2 as points in blue, orange, and purple, respectively. Importantly, the MC simulation results are in quantitative agreement with experimental FRAP data taken from Reference [9], which validates our computational model.

The data presented in Figure 2 clearly demonstrate that the binding affinity $K_{2D}$ is not constant but rather increases with the area concentration [RL] of CD47–SIRPα complexes. Thus, the more CD47–SIRPα complexes are formed, the larger the CD47–SIRPα binding affinity gets, which implies that increasing the amount of CD47–SIRPα complexes facilitates the formation of extra CD47–SIRPα complexes. Therefore, CD47–SIRPα binding is a cooperative process. The cause of this binding cooperativity is that the formation of CD47–SIRPα complexes smoothens membrane fluctuations, which, in turn, facilitates the formation of additional CD47–SIRPα complexes [6,8,9].

**Figure 3:**
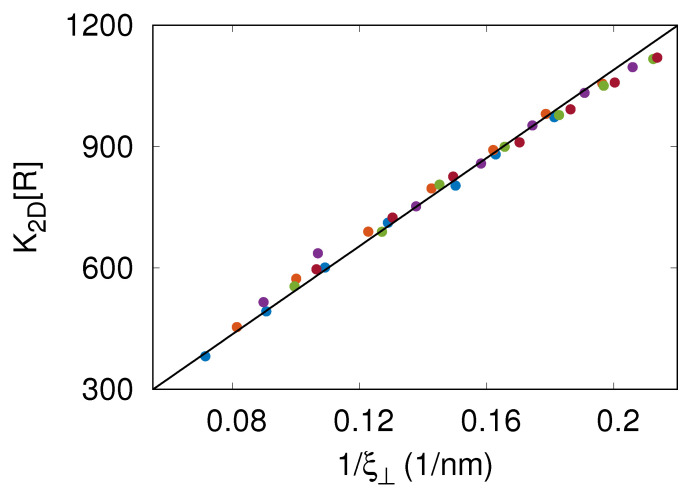
Two-dimensional binding affinity $K_{2D}$ times the area concentration of receptors, [RL]=4000/μm${}^{2}$, versus the thermal roughness $\xi_{⊥}$ of the membrane. The different colors represent the results of MC simulations with $l_{b}=5.4,\;6.6,\ldots 9,\;10.2$ nm. The solid line in black shows the relation $K_{2D}\left[\mathrm{RL}\right]=\ell_{1}/\xi_{⊥}$ with $\ell_{1}=5.45\;\mu m$ being a fitting parameter. This relation is equivalent to $K_{2D}/K_{3D}=c_{1}/\xi_{⊥}$ with $c_{1}=1.22$, where $1/K_{3D}=1\;\mu M$ is the dissociation constant of the soluble variants of CD47 and SIRPα.

In the MC simulations, we also measured the thermal roughness $\xi_{⊥}$ of the membrane. This roughness results from thermally excited undulations of the flexible membrane. Figure 3 shows that the results of MC simulations with $l_{b}=5.4,\;6.6,\ldots 9,\;10.2$ nm overlie on a master curve $K_{2D}\left[RL\right]=\ell_{1}/\xi_{⊥}$ with $\ell_{1}=5.45\;\mu m$. Since [RL]≈4000/μm${}^{2}$ [9] and the dissociation constant of the soluble variants of CD47 and SIRPα is $1/K_{3D}\approx 1\;\mu M$ [1], the latter relation is equivalent to $K_{2D}/K_{3D}=c_{1}/\xi_{⊥}$ with a dimensionless coefficient $c_{1}=1.22$. The relation $K_{2D}/K_{3D}∼1/\xi_{⊥}$ has been observed previously in DPD simulations of a generic, coarse-grained molecular model [6].

**Figure 4:**
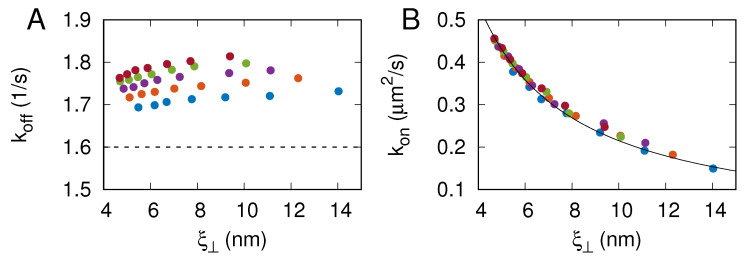
CD47–SIRPα binding rate constants $k_{\mathrm{off}}$ (**A**) and $k_{\mathrm{on}}$ (**B**) as a function of membrane roughness $\xi_{⊥}$. The different colors represent the results of MC simulations with $l_{b}=5.4,\;6.6,\ldots 9,\;10.2$ nm. The dashed line in panel (**A**) indicates $k_{\mathrm{off}}=1.6$ s${}^{-1}$, determined in surface plasmon resonance experiments [14]. The solid line in panel (**B**) shows $k_{\mathrm{on}}=c_{2}/\xi_{⊥}$ with $c_{2}$ being a fit parameter.

MC simulations with local moves can be used to study membrane dynamics in the overdamped limit [23,25]. Here, we keep track of receptor–ligand binding and unbinding events in the course of the MC simulations. The maximum likelihood method used to extract the binding rate constants $k_{\mathrm{on}}$ and $k_{\mathrm{off}}$ from the MC simulation trajectories is detailed in Section 2.2. Figure 4A shows that the $k_{\mathrm{off}}$ values obtained from the simulations are in the range between 1.7 and 1.8 s${}^{-1}$. These values indicate that the off-rate is reaction-limited because $\ln\left(k_{\mathrm{off}}\;\tau \right)\approx -U/k_{B}T$, where τ=60μs is the simulation step time and $U=9\;k_{B}T$ is the depth of the receptor–ligand binding potential given by Equation (2). Importantly, the $k_{\mathrm{off}}$ values obtained from the simulations compare well with $k_{\mathrm{off}}=1.6$ s${}^{-1}$ measured in surface plasmon resonance experiments [14], which additionally validates our computational model because no kinetic information is incorporated into the construction of the model.

As can be seen in Figure 4A, the CD47–SIRPα dissociation rate constant $k_{\mathrm{off}}$ does not exhibit any particular dependence on the membrane roughness $\xi_{⊥}$ and varies only very weakly with $l_{b}$. In contrast, the CD47–SIRPα association rate constant $k_{\mathrm{on}}$ decreases monotonically with the membrane roughness $\xi_{⊥}$, as can be seen in Figure 4B. In fact, the data points obtained from the simulations with $l_{b}=5.4,\;6.6,\;\ldots 9,\;10.2$ nm overlie on a master curve $k_{\mathrm{on}}=c_{2}/\xi_{⊥}$ with $c_{2}=2.15$ nm${}^{3}$/μs. The relation $k_{\mathrm{on}}∼\xi_{⊥}$ is not fully consistent with the results reported by Hu et al. [6], probably because the relatively fast off-rates in the DPD simulations were not reaction-limited.

**Figure 5:**
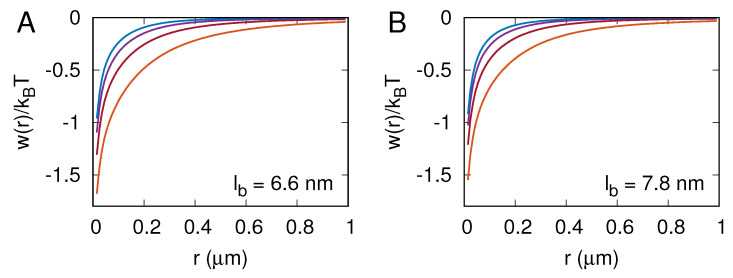
Potential of mean force $w\left(r\right)=-k_{B}T\ln g\left(r\right)$, where g(r) is the two-dimensional pair correlation function for receptor–ligand complexes. Panels (**A**,**B**) correspond to $l_{b}=7.8$ nm and $l_{b}=6.6$ nm, respectively. The lines in orange, red, purple, and blue correspond to 30, 50, 70, and 90 receptor–ligand complexes per square micron.

In the MC simulations, we also computed the two-dimensional pair correlation function g(r) for receptor–ligand complexes and the corresponding potential of mean force $w\left(r\right)=-k_{B}T\ln g\left(r\right)$. Panels A and B in Figure 5 show the potential of mean force w(r) for $l_{b}=7.8$ nm and $l_{b}=6.6$ nm, respectively. The lines in orange, red, purple, and blue correspond to [RL]=30, 50, 70, and 90 per μ$m^{2}$. Importantly, w(r)<0 and ∂w/∂r>0 in all of the cases studied here, which means that the receptor–ligand complexes are always effectively attracted one to another. This effective attraction between the receptor–ligand complexes is rather weak ($\left|w(r)\right|<2k_{B}T$) and has a very long range, as it vanishes on the length scale of a micrometer.

The membrane-mediated attraction between receptor–ligand complexes, as quantified here with the potential of mean force w(r), is entropic in nature and originates from the suppression of conformational fluctuations of the membrane by receptor–ligand complexes [26,27,28]. It can be seen in Figure 5 that this attraction is strongest at the lowest receptor–ligand concentration, [RL]=30μm${}^{2}$, which makes sense because conformational fluctuations of the membrane are largest in that case. The graphs of w(r) in Figure 5 also show that both the magnitude and the range of the effective attraction decrease with increasing the area concentration of receptor–ligand complexes, which is reasonable because the more receptor–ligand complexes are formed, the weaker the membrane fluctuates.

Based on the potential of mean force, w(r), we computed the two-dimensional second virial coefficient [29]

(12) $$B_{2}=-\pi \int_{0}^{\infty }\left[e^{-w\left(r\right)/k_{B}T}-1\right]rdr\;.$$

**Figure 6:**
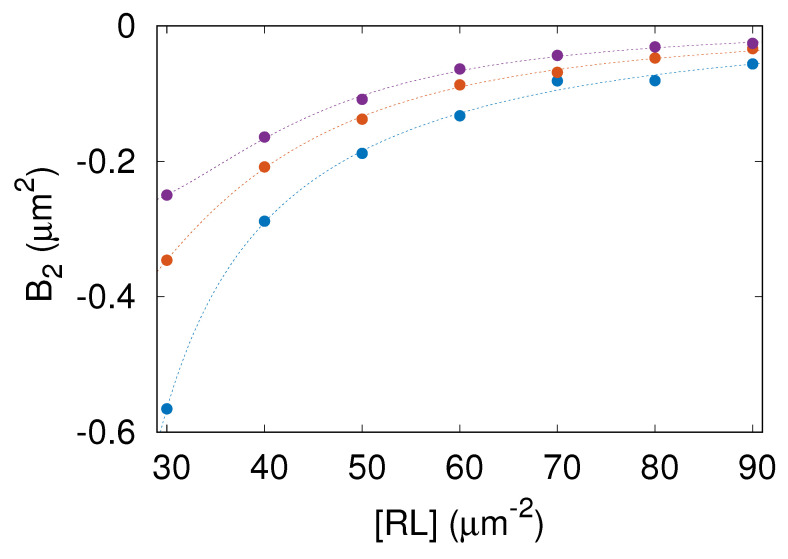
Second virial coefficient, $B_{2}$, as a function of the area concentration of the receptor–ligand complexes, [RL], computed for $l_{b}=5.4$ nm (blue), 6.6 (orange), and 7.8 (purple). The color code and symbols are as in Figure 2.

Figure 6 shows the computed values of $B_{2}$ versus [RL] for $l_{b}=5.4,\;6.6$, and 7.8 nm. The color code is as in Figure 2. Importantly, $B_{2}<0$ in the whole range of parameters studied here. The negative values of $B_{2}$ mean that the receptor–ligand complexes are effectively attracted one to another. More negative values of $B_{2}$ imply stronger effective attraction between the receptor–ligand complexes.

The lowest value of $B_{2}$ found in the parameter range studied here is about −0.6μm${}^{2}$. As can be seen in Figure 6, $B_{2}$ increases with both [RL] and $l_{b}$. Consequently, the membrane-mediated attraction between receptor–ligand complexes is strongest for $l_{b}=5.4$ nm and gets weaker as $l_{b}$ is increased. This result is understandable because conformational fluctuations of the membrane are suppressed to a larger extent by receptor–ligand complexes when $l_{b}$ is smaller.

The effective, fluctuation-induced, membrane-mediated attraction between receptor–ligand complexes is not strong enough to induce phase separation within the membrane. Generally, if the adhesion of tensionless membranes is mediated by only one type of receptor–ligand complex, as in the system studied here, additional interactions (such as direct attraction between adhesion molecules [21] or generic repulsion between the apposing membranes [22] or the association of adhesion molecules with lipid rafts [11,12]) are necessary for separation between a phase depleted of receptors and a phase enriched in receptor–ligand complexes. However, the negative values of the second virial coefficient $B_{2}$ found in this study reveal a propensity of CD47–SIRPα complexes to form transient clusters.

## 4. Discussion

The simulations presented here allowed us to capture processes occurring at various length scales, ranging from the specific receptor–ligand binding at the distance $l_{\mathrm{we}}=1.2$ nm all the way up to membrane elastic deformations at the length comparable to the simulation box size L=6μm. These processes were simulated on the time scale of about one hour. Importantly, the simulations not only reproduced the dependence of $K_{2D}$ on [RL] obtained in the fluorescence microscopy experiments [9] (Figure 2) but also yielded the CD47–SIRPα dissociation rate constant consistent with the $k_{\mathrm{off}}$ value determined via surface plasmon resonance [14] (Figure 4A). The simulation results also complied with the general relationship $K_{2D}∼1/\xi_{⊥}$ derived by Hu et al. [6] (Figure 3) and demonstrated that the CD47–SIRPα association rate constant $k_{\mathrm{on}}∼1/\xi_{⊥}$ (Figure 4B).

The CD47–SIRPα complex concentration [RL] and binding affinity $K_{2D}$ are found to be positively correlated (Figure 2). This means that the more CD47–SIRPα complexes are formed in the adhesion zone, the larger the CD47–SIRPα binding affinity gets, which implies that increasing the number of CD47–SIRPα complexes leads to the formation of extra CD47–SIRPα complexes. Therefore, CD47–SIRPα binding is a cooperative process.

The CD47–SIRPα binding cooperativity is due to thermal fluctuations and elastic properties of the membrane. Namely, the GPMV membrane is rather soft (its bending rigidity modulus $\kappa \approx 10\;k_{B}T$) and undergoes thermally excited fluctuations, which is reflected in the membrane roughness $\xi_{⊥}$ up to 14 nm (Figure 3). As CD47–SIRPα complexes are formed, fluctuations in the local distance between the membrane and the planar surface are suppressed and, thus, the membrane roughness $\xi_{⊥}$ decreases. Then, the CD47 molecules anchored in the membrane are more likely to be present in the binding distance from the surface-immobilized SIRPα molecules, which facilitates formation of additional CD47–SIRPα complexes. Indeed, the binding affinity $K_{2D}$ is found to increase as the membrane roughness $\xi_{⊥}$ is suppressed due to increasing the CD47–SIRPα complex concentration [RL] (Figure 3).

Our analysis of the simulation data revealed long-ranged, membrane-mediated, entropic attraction between CD47–SIRPα complexes. To explain the origin of this attraction, we note that the number of membrane conformations is larger when many CD47–SIRPα complexes form one cluster and act effectively as one constraint on the local distance between the membrane and the surface than when CD47–SIRPα complexes are distributed more-or-less uniformly and act as multiple constraints on the local distance between the membrane and the surface. Thus, clustering CD47–SIRPα complexes decreases the entropy of the adhered membrane, which is the cause of the membrane-mediated, entropic attraction between CD47–SIRPα complexes. This type of effect has been studied theoretically using generic models [27] and demonstrated in experiments on cadherin-mediated adhesion [28]. To the best of our knowledge, however, membrane-mediated interactions between CD47–SIRPα complexes have not been determined so far. Here, we quantified the membrane-mediated, effective attraction between CD47–SIRPα complexes in terms of the potential of mean force (Figure 5), which adds to the novelty of our study. Moreover, we determined the second virial coefficient as a function of the area concentration of CD47–SIRPα complexes (Figure 6). Apparently, such detailed information on indirect interactions between CD47–SIRPα complexes cannot currently be obtained from experiments alone. Our approach combining physics-based computer simulations with available experimental data is unique and provides new insights into the interactions between CD47 and SIRPα.

The CD47–SIRPα innate immune checkpoint has been in the focus of biomedical research [30,31,32,33,34]. The binding of CD47 to SIRPα has been found to play important roles in phagocytosis, auto-immunity, and host defense [1,2]. As such, the CD47–SIRPα protein complex has been recognized as a potential therapeutic target in cancer [3,4,30,32,33,35] and inflammation [5]. Our simulation results extend the present understanding of cooperative effects in CD47–SIRPα interactions and thus can influence advancements of new cancer treatments [4,35].

It is important to note that the lattice-based mesoscale model employed in this study has several limitations. First of all, as CD47 and SIRPα molecules are represented by single particles with no internal structure, the conformational and rotational entropy of these proteins is not included in the model. Secondly, the discretization of the membrane into square patches can impose artifacts in the distribution of the ligands within the membrane. It also limits the spatial resolution in the membrane lateral directions to a=15 nm and the temporal resolution to about 60 μs. In principle, all of the aforementioned limitations can be overcome by using coarse-grained molecular dynamics simulations [6,36,37]. However, the computational costs still prohibit coarse-grained molecular dynamics simulations from exploring the length and time scales investigated in the present study using a lattice-based mesoscale model.

The major advantage of our present work over previous studies on the cooperative binding of CD47 with SIRPα [9] is that, here, we captured and quantified the long-ranged, membrane-mediated, entropic attraction between CD47–SIRPα complexes (Figure 5 and Figure 6). We also established how membrane fluctuations affect the CD47–SIRPα binding rate constants $k_{\mathrm{on}}$ and $k_{\mathrm{off}}$ (Figure 4). This progress was possible because we carefully parameterized a lattice-based mesoscale model, performed extensive simulations of a sufficiently large adhesion zone with the linear size L=6μm, and analyzed the simulation data in detail to determine various physical quantities. Further insights into the dynamics of the CD47–SIRPα checkpoint can be gained in the future using coarse-grained molecular dynamics simulations [36,37].

## Data Availability

The data presented in this study are openly available in [Monte Carlo Simulations of the Membrane-Mediated Cooperative Interactions between CD47 and SIRP] at [https://doi.org/10.18150/RLZZSW].
